# Sarcomatoid (spindle cell) carcinoma of the pancreas: A case report and review of the literature

**DOI:** 10.3892/ol.2013.1683

**Published:** 2013-11-14

**Authors:** JOSHUA ROBERT KANE, WILLIAM B. LASKIN, KRISTINA A. MATKOWSKYJ, CELINA VILLA, ANJANA V. YELDANDI

**Affiliations:** Department of Pathology, Northwestern University, Feinberg School of Medicine, Chicago, IL 60611, USA

**Keywords:** sarcomatoid carcinoma, carcinosarcoma, pancreas, spindle cell carcinoma, immunohistochemistry

## Abstract

Sarcomatoid (spindle cell) carcinoma of the pancreas is a rare, high-grade epithelial malignancy composed predominantly or exclusively of spindle cells demonstrating evidence of epithelial derivation, but no features indicative of a specific line of mesenchymal differentiation. The current study presents the case of an 85-year-old Caucasian male with a tumor mass in the body of the pancreas. The individual subsequently underwent a distal pancreatectomy, splenectomy and partial gastrectomy. Microscopic examination of the 3.3-cm mass located in the body of the pancreas revealed a small, but high-grade, adenocarcinomatous component that blended imperceptibly with malignant spindle cells. No light microscopic or immunohistochemical evidence of specific mesenchymal differentiation was identified, and the spindle cells, as in the case of the carcinoma, were diffusely keratin-positive. Sarcomatoid (spindle cell) carcinoma defined in this way rarely presents in the pancreas, with, to the best of our knowledge, only six cases reported in the English literature.

## Introduction

Sarcomatoid (spindle cell) carcinoma is an aggressive form of carcinoma composed of malignant spindle cells, with or without a coexisting epithelial cell component. The carcinoma demonstrates evidence of epithelial derivation with no specific line of mesenchymal differentiation. The current study represents only the seventh case of sarcomatoid (spindle cell) carcinoma of the exocrine pancreas as defined above that has described in the English literature (1–6).

## Case report

### Materials and methods

Tissue from the pancreatic tumor was obtained from the surgical pathology files of Northwestern Memorial Hospital (Chicago, IL, USA). Light microscopic and immunohistochemical examination were performed on hematoxylin and eosin-stained sections prepared from formalin-fixed, paraffin-embedded tissue. The immunomarkers used, including clones, dilutions and manufacturers, are presented in Table I. Cases relevant to the definition of sarcomatoid (spindle cell) carcinoma reported in the English literature are listed in Table II and were searched for via PubMed.

### Patient presentation and diagnosis

The need for written consent was waived by the Institutional Review Board of Northwestern University (Chicago, IL, USA). An 85-year-old Caucasian male presented to Northwestern Memorial Hospital (Chicago, IL, USA) with signs and symptoms resembling earlier episodes of pancreatitis that had been experienced over the past 8 months. Endoscopic ultrasound identified a well-circumscribed, hypoechoic mass adjacent to the portal vein within the pancreatic body. A pre- and post-contrast helical abdominal (pancreatic and portal venous phase) and pelvic (venous phase) CT demonstrated a unilocular, non-enhancing, cystic mass measuring 3.7×2.7 cm that obstructed the main pancreatic duct within the body of the pancreas. The mass was homogeneously enhanced and exhibited diffuse peripancreatic stranding. According to these radiological observations, an initial clinical diagnosis of an adenocarcinoma or neuroendocrine tumor was formed. A fine-needle aspiration of the mass was performed prior to surgery and revealed high-grade malignant epithelial cells in a pseudopapillary pattern. A second population of more primitive tumor cells was identified with high nuclear/cytoplasmic ratios within a richly mucinous stromal background. In addition, laparoscopic distal (near-total) pancreatectomy, splenectomy and partial gastrectomy were performed. The patient was alive and well 26 months after the surgery.

### Pathological observations

**Figure 1:**
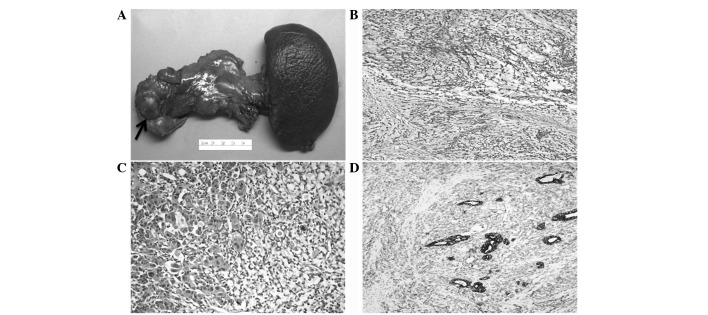
(A) Intact speciman consisting of the pancreatic body and tail with the attached spleen and portion of stomach. The tumor nodule is indicated by the arrow. (B) Spindle cell component of the tumor with background myxoid matrix (H&E; magnification, ×10). (C) Scattered malignant epithelial cells merged imperceptibly with the cytologically atypical spindle cells. A scant cytoplasm and no distinct features of specific mesenchymal differentiation were identified. (H&E; magnification, ×40) (D) Keratin staining (MNF1) was positive in the glandular and spindle cell elements of the tumor (magnification, ×10).

The surgical specimen consisted of the pancreatic body and tail with the attached spleen and a portion of the stomach (Fig. 1A). The cut surface of the body of the pancreas revealed a poorly-circumscribed, solid, fleshy mass of variegated yellow-tan to dark red color, measuring 3.3×3.0×2.6 cm. The tumor mass was adherent to the serosa of the stomach, adjacent to the splenic artery and vein and externally compressed and obstructed the main pancreatic duct (Fig. 1A).

Ill-defined, highly cellular nodules of cytologically atypical spindle cells, intermingling with scattered adenocarcinomatous elements within a myxoid stroma were determined by scanning light microscopy (Fig. 1B). In addition, the lesional tissue surrounded a central area of necrosis. At higher magnification, the minor adenocarcinomatous component, including ill-defined ductal structures and individually dispersed cells, blended imperceptibly with a haphazardly arranged, cellular proliferation of cytologically atypical spindle cells. The cells exhibited a mild to moderate degree of nuclear pleomorphism and scanty eosinophilic cytoplasm. However, no microscopic features indicating specific mesenchymal differentiation were identified (Fig. 1C). Mitotic figures were readily identified in the two components. Perineural invasion and regional lymph node metastasis by the spindle component were evident, but lymphovascular invasion was not identified. The surrounding non-neoplastic parenchyma exhibited features of chronic pancreatitis. The immunohistochemical results are presented in Table I and indicate that the epithelial and spindle tumor cells diffusely expressed pan-cytokeratin MNF-116 (Fig. 1D), cytokeratin 8/18 and nuclear p53, but not nuclear β-catenin, calponin, CD10 or p63. Synaptophysin, chromogranin and CD56 were expressed in scattered lesional cells and Ki-67 immunoexpression was demonstrated in >50% of malignant cells. According to the histological and immunohistochemical observations, a diagnosis of primary sarcomatoid (spindle cell) carcinoma of the pancreas was formed.

## Discussion

Sarcomatoid (spindle cell) carcinoma is characterized by malignant spindle cell proliferation that demonstrates epithelial derivation, but no light microscopic, ultrastructural or immunohistochemical evidence indicating a specific line of mesenchymal differentiation. The microscopically nondescript spindle cells, similar to the epithelial component, typically express keratin or other epithelial-related markers, albeit often in a focal manner, or exhibit other attributes consistent with an epithelial pathogenesis. Carcinosarcoma is an epithelial malignancy associated with sarcomatoid (spindle cell) carcinoma with an equally aggressive clinical course that by definition demonstrates biphasic epithelial and mesenchymal differentiation (7). Therefore, for practical diagnostic purposes, carcinosarcoma is used interchangeably with sarcomatoid (spindle cell) carcinoma. In the present case report, carcinosarcomas with heterologous mesenchymal elements demonstrating light microscopic and/or immunohistochemical evidence of specific mesenchymal (lipogenic, smooth or skeletal muscle, peripheral nerve sheath, vascular or osteo-/cartilaginous) differentiation were excluded. However, the World Health Organization classification of exocrine pancreatic tumors allocates spindle cell carcinoma, sarcomatoid carcinoma and carcinosarcoma under the rubric of undifferentiated (anaplastic) carcinoma (8), since the majority of these types of tumor possess a spindle element that demonstrates an epithelial immunohistochemical profile and/or genetic alterations in pancreatic ductal adenocarcinomas (7).

To date, the largest study to analyze the histological spectrum of sarcomatoid carcinoma, including examples of malignant pancreatic neoplasms exhibiting varied sarcoma-like features, was reported by Alguacil-Garcia *et al*(9). This study identified four distinctive histological types of sarcomatoid carcinoma based on light microscopic analysis only. The four subtypes were as follows: i) Spindle cell carcinoma, consisting primarily of malignant spindle cells; ii) osteoclastic giant cell tumors, demonstrating an admixture of malignant spindle and epithelioid-appearing cells with osteoclast-like giant cells; iii) pleomorphic giant cell carcinoma, exhibiting highly pleomorphic mononuclear and multinucleated giant cells; and iv) round cell anaplastic carcinoma, composed exclusively of monotonous rounded, small- to moderately-sized tumor cells. The reported prognosis of each of these histological subtypes was uniformly poor (9), consistent with the aggressive nature of this subset of malignant tumors.

However, it is difficult to classify tumors belonging to any of these four categories as spindle cell carcinoma or carcinosarcoma with heterologous elements, as aforementioned, or even to exclude pure sarcoma or carcinoma in specific cases without the benefit of immunohistochemistry, electron microscopy or molecular studies. In retrospect, we hypothesize that the spindle cell variant most closely resembles the tumor identified in the patient discussed in the present case report. The pleomorphic epithelioid cells that typify pleomorphic giant cell carcinoma have the potential for focal transition to a spindle morphology. However, this neoplasm must not exhibit a distinct spindle cell component like the sarcomatoid (spindle cell) carcinoma or carcinosarcoma. The majority of pancreatic tumors with osteoclastic giant cells are epithelial tumors with accompanying pleomorphic giant cells (pleomorphic/osteoclastic or ‘mixed’ giant cell tumor) (10) or tumors histologically resembling giant cell tumors of the bone (osteoclastic giant cell tumor or osteoclastoma) (11), but whose immunohistochemical and molecular profiling are consistent with an epithelial derivation (12). In contrast to the malignant mononuclear cell element of the tumor presented, previous studies have shown that the osteoclastic giant cell is a benign component of monocytic derivation recruited into the lesional cell environment via the production of cytokines by neoplastic cells (13,14).

In addition to the tumor of the present case study, six additional examples of pancreatic sarcomatoid (spindle cell) carcinoma with confirmed epithelial derivation of the spindle component and/or absence of specific mesenchymal differentiation have been identified in a comprehensive review of the English literature (PubMed; Table II) (1–6). Of the patients with adequate follow-up, 4/5 succumbed to their condition within 9 months of surgery (1,4–6). In a previous review of pancreatic tumors classified as ‘carcinosarcomas’, including two cases that are included in the present case report as examples of sarcomatoid (spindle cell) carcinoma (2,3), Gelos *et al*(15) identified that the average post-operative survival interval was 6 months and that the longest living patient survived for 15 months. Notably, the patient of the present case study was alive and well 26 months after surgery and thus, to the best of our knowledge, is the longest-living individual with pancreatic sarcomatoid carcinoma and carcinosarcoma.

Consistent with the results of the present case report, an epithelial origin of the spindle cell element has been identified by the presence of epithelial immunomarkers, including keratin(s) and EMA, within the spindle cell component in five of the six reported pancreatic sarcomatoid carcinomas (1–5). Higashi *et al*(1) also reported the immunoexpression of MUC1, an apomucin core protein more specific than keratin for pancreatic ductal carcinoma, within malignant spindle cells of pancreatic sarcomatoid (spindle cell) carcinoma.

Molecular analysis is a useful technique for establishing a diagnosis of sarcomatoid (spindle cell) carcinoma by demonstrating a histogenetic link between malignant spindle cells and pancreatic ductal adenocarcinoma. The K-ras mutation at codon 12 of exon 2 is implicated in the pathogenesis of pancreatic ductal adenocarcinoma (16) and has been identified in the spindle cell component of two examples of pancreatic sarcomatoid (spindle cell) carcinoma. Nakano *et al*(5) identified a mutation in the keratin-expressing spindle component of a malignant biphasic pancreatic tumor. Kim *et al*(6) identified the mutation in the epithelial and spindle elements of a malignant biphasic pancreatic tumor. Although the spindle component failed to express keratin, a broad mesenchymal immunoprofile was also negative. These two cases are consistent with the current belief that the vast majority of malignant spindle cell neoplasms of the pancreas are sarcomatoid carcinomas. In addition, the observation of the K-ras mutation at codon 12 of exon 2 within a retroperitoneal-based, undifferentiated spindle cell malignancy is likely to indicate a pancreatic epithelial origin, particularly in cases where there is no evidence of a coexisting or preexisting well-differentiated component (eliminating ‘dedifferentiated’ sarcoma) and where the tumor lacks specific mesenchymal or epithelial immunomarker expression.

The pathogenesis of sarcomatoid malignant biphasic neoplasms remains unclear, but certain previous studies indicate a monoclonal (stem cell) origin with divergence into carcinoma and sarcomatous elements (17,18). Epithelial-to-mesenchymal transition has been postulated to be an alternative mechanism explaining the origin of a sarcomatous component from a carcinoma based on the differential loss or gain of genes between the two components (5,19). The identification of the characteristic K-ras mutation in codon 12 of exon 2 in a malignant spindle cell pancreatic tumor constitutes evidence of a monoclonal origin of the two components of sarcomatoid carcinoma. Nakano *et al*(5) postulated that an additional K-ras mutation at codon 34 of exon 2, located within the spindle cell element, facilitated an epithelial-to-mesenchymal (sarcomatous) transformation. Using an alternative approach to demonstrate monoclonality, van den Berg *et al*(18) identified identical genetic aberrations in six chromosomal loci. The loci are commonly altered in the two cellular elements of pancreatic ductal carcinoma in cases of pancreatic mucinous neoplasm with a coexisting sarcomatous stroma that lack keratin immunoexpression and show morphological and/or immunohistochemical evidence of mesenchymal differentiation.

In conclusion, the current case study documents the seventh case of sarcomatoid (spindle cell) carcinoma of the pancreas with data substantiating the epithelial derivation of the nondescript malignant spindle cell element. The patient presented here was alive and disease-free at 26 months post-surgery, and is therefore the longest disease-free survivor reported in the English language literature.

## Figures and Tables

**Table I:** Immunohistochemical findings.

Antibody	Reactivity	Clone	Dilution	Source
Calponin	−	CALP	1:400	Dako^a^
CD10	F+	56C6	1:30	Leica^b^
Chromogranin	−	LK2H10	Predilute	Ventana Medical Systems^c^
CK8/18	D+	CAM5.2	1:50	Becton-Dickinson^d^
CK19	−	RCK108	1:100	Dako^a^
EMA	−	E29	1:50	Dako^a^
Ki-67	50%	30-9	Predilute	Ventana Medical Systems^c^
MUC1	−	Ma695	1:100	Vector Laboratories^e^
Nuclear β-catenin	−	14	1:200	Becton-Dickinson^d^
p53	75%	Bp-53-11	Predilute	Ventana Medical Systems^c^
p63	−	4A4	Predilute	Ventana Medical Systems^c^
Pan-CK	D+	MNF116	1:50	Dako^a^
S100	−	4C4.9	Predilute	Ventana Medical Systems^c^
SMA	−	1A4	Predilute	Cell Marque Corporation^f^
Synaptophysin	−	N/A	1:50	Cell Marque Corporation^f^
Vimentin	D+	V9	1:100	Dako^a^

a Dako, Carpinteria, CA, USA;

b Leica, Mannheim, Germany;

c Ventana Medical Systems, Inc., Oro Valley, AZ, USA;

d Becton-Dickinson, San Jose, CA, USA;

e Vector Laboratories, Burlingame, CA, USA;

f Cell Marque Corporation, Rocklin, CA, USA.

CK, cytokeratin; EMA, epithelial membrane antigen; SMA, smooth muscle actin; D+, diffuse positivity; F+, focal positivity; −, negative.

**Table II:** Clinical and pathological observations in seven cases of sarcomatoid spindle cell (SC) carcinoma of the pancreas reported in the English literature.

First author, year (ref.)	Age, years/gender	Tumor size, cm	Carcinoma	Sarcomatoid component	Molecular	Follow-up, months/outcome
Higashi *et al*, 1999 (1)	74/male	4.5×4.0×3.0	PD adeno	SC; IHC: CK AE1, variable CK AE3, EMA, MUC1-ARA (D+), S100, SMA (F+), desmin, vimentin, NSE and CEA (−)	NA	3/succumbed to diffuse peritoneal carcinomatosis
Darvishian *et al*, 2001 (2)	74/male	4.0×3.0	MD adeno	SC; IHC:vimentin (D+), CK (F+), CEA, SMA, desmin and CD68 (−)	NA	4/alive and well
Barkatullah *et al*, 2005 (3)	67/female	2.5×2.5×2.0	MD adeno	SC, separate focus of OGC; IHC (SC): CK8/18 and vimentin (D+)	NA	8/NA
De la Riva *et al,* 2006 (4)	72/female	NA	Not identified, but associated with choledochal cyst	SC; IHC: CK and vimentin (F+)	NA	9/succumbed to sarcomatoid carcinoma metastatic to the liver
Nakano *et al,* 2007 (5)	82/female	18.0×11.0×10.0	WD adeno	SC, foci of OGC around hemorrhage; IHC (SC): vimentin, CD10 (D+), CK AE1/AE3 (F+), CK7, CK20, CEA, EMA, SMA and S100 (−)	K-ras mutation at codon 12 (and codon 34) of exon 2 in SC	0/Succumbed to DIC on post-operative day 13
Kim *et al*, 2010 (6)	48/male	3.5×2.5×1.5	Mucinous cyst adeno and anaplastic carcinoma	SC, scattered OGC; IHC (SC): vimentin (D+), pan-CK, CK, 7, CK8/18, EMA, CEA, CD34, CD56, CD68, CD117, desmin, SMA, myogenin, S100, ER and PR (−)	K-ras mutation at codon 12 of exon 2 in SC and epithelial components	4/succumbed to hepatic and peritoneal metastases
Current case report, 2013	85/male	3.3×3.0×2.6	PD adeno	SC; IHC: diffuse pan-CK, CK5.2, p53 (D+), synaptophysin, chromogranin, calponin, S100, SMA, CK19, MUC1, nuclear β-Catenin, p63, EMA and CD10 (−)	NA	26/alive and well

PD, poorly-differentiated; adeno, adenocarcinoma; IHC, immunohistochemistry; CK, cytokeratin; EMA, epithelial membrane antigen; MUC1-ARA, apoprotein MUC1; (D+), diffusely positive; SMA, smooth muscle actin; (F+), focal positivity; NSE, neuron-specific enolase; CEA, carcinoembryonic antigen; (−), no positivity; NA, data not available; MD, moderately-differentiated; OCG, osteoclastic giant cells; WD, well-differentiated; DIC, disseminated intravascular coagulopathy; ER, estrogen receptor protein; PR, progesterone receptor protein.

## References

[1] Higashi M, Takao S, Sato E (1999). Sarcomatoid carcinoma of the pancreas: a case report with immunohistochemical study. Pathol Int.

[2] Darvishian F, Sullivan J, Teichberg S, Basham K (2002). Carcinosarcoma of the pancreas: a case report and review of the literature. Arch Pathol Lab Med.

[3] Barkatullah SA, Deziel DJ, Jakate SM, Kluskens L, Komanduri S (2005). Pancreatic carcinosarcoma with unique triphasic histological pattern. Pancreas.

[4] De la Riva S, Muñoz-Navas MA, Betés M, Súbtil JC, Carretero C, Sola JJ (2006). Sarcomatoid carcinoma of the pancreas and congenital choledochal cyst. Gastrointest Endosc.

[5] Nakano T, Sonobe H, Usui T, Yamanaka K, Ishizuka T, Nishimura E, Hanazaki K (2008). Immunohistochemistry and K-ras sequence of pancreatic carcinosarcoma. Pathol Int.

[6] Kim HS, Joo SH, Yang DM, Lee SH, Choi SH, Lim SJ (2011). Carcinosarcoma of the pancreas: a unique case with emphasis on metaplastic transformation and the presence of undifferentiated pleomorphic high-grade sarcoma. J Gastrointestin Liver Dis.

[7] Hruban RH, Pitman MB, Klimstra DS (2007). Adenocarcinoma variants. Tumors of the Pancreas AFIP atlas of tumor pathology: Fourth series.

[8] Fukushima N, Hruban RH, Kato Y, Klimstra DS, Kloppel G, Shimizu M, Terris B, Bosman FT, Carneiro F, Hruban RH, Theise ND (2010). Ductal adenocarcinoma variants and mixed neoplasms of the pancreas. World Health Organization Classification of Tumours of the Digestive System.

[9] Alguacil-Garcia A, Weiland LH (1977). The histologic spectrum, prognosis, and histogenesis of the sarcomatoid carcinoma of the pancreas. Cancer.

[10] Watanabe M, Miura H, Inoue H, Uzuki M, Noda Y, Fujita N, Yamazaki T, Sawai T (1997). Mixed osteoclastic/pleomorphic-type giant cell tumor of the pancreas with ductal adenocarcinoma: histochemical and immunohistochemical study with review of the literature. Pancreas.

[11] Rosai J (1968). Carcinoma of pancreas simulating giant cell tumor of bone. Electron-microscopic evidence of its acinar cell origin. Cancer.

[12] Westra WH, Strum P, Drillenburg P, Choti MA, Klimstra DS, Albores-Saavadra J (1998). K-ras oncogene mutations in osteoclast-like giant cell tumors of the pancreas and liver: genetic evidence to support origin from the duct epithelium. Am J Surg Pathol.

[13] Goldberg RD, Michelassi F, Montag AG (1991). Osteoclast-like giant cell tumor of the pancreas: immunophenotypic similarity to giant cell tumor of bone. Hum Pathol.

[14] Leighton CC, Shum DT (2001). Osteoclastic giant cell tumor of the pancreas: case report and literature review. Am J Clin Oncol.

[15] Gelos M, Behringer D, Philippou S, Mann B (2008). Pancreatic carcinosarcoma. Case report of multimodal therapy and review of the literature. JOP.

[16] Almoguera C, Shibata D, Forrester K, Martin J, Arnheim N, Perucho M (1988). Most human carcinomas of the exocrine pancreas contain mutant c-K-ras genes. Cell.

[17] Thompson L, Chang B, Barsky S (1996). Monoclonal origins of malignant mixed tumors (carcinosarcomas). Evidence for a divergent histogenesis. Am J Surg Pathol.

[18] van den Berg W, Tascilar M, Offerhaus GJ (2000). Pancreatic mucinous cystic neoplasms with sarcomatous stroma: molecular evidence for monoclonal origin with subsequent divergence of the epithelial and sarcomatous components. Mod Pathol.

[19] Zhuang ZP, Lininger RA, Man YG, Albuquerque A, Merino MJ, Tavassoli FA (1997). Identical clonality of both components of mammary carcinosarcoma with differential loss of heterozygosity. Mod Pathol.

